# Can educational video resources improve learning when used to augment traditional teaching of clinical examination? A randomized control trial of novice medical students

**DOI:** 10.1186/s12909-022-03974-8

**Published:** 2023-01-12

**Authors:** Ellie Flatt, Paul Brewer, Malek Racy, Faisal Mushtaq, Rachael Ashworth, Fazal Ali, James Tomlinson

**Affiliations:** 1 Sheffield Teaching Hospitals, Northern General Hospital, Herries Road, Sheffield, S5 7AU UK; 2 University of Sheffield, Western Bank, Sheffield, S10 2TN UK; 3 School of Psychology, Faculty of Medicine and Health, University of Leeds, Leeds, LS2 9JT UK; 4 Hull University Teaching Hospitals, Hull Royal Infirmary, Anlaby Road, Hull, HU3 2JZ UK; 5 Chesterfield Royal Hospital, Chesterfield Road, Chesterfield, S44 5BL, Chesterfield, UK

**Keywords:** Physical examination, Medical education, Educational technology, Distance learning

## Abstract

**Background:**

Good clinical examination skills can both increase the quality of patient care and reduce its cost. A previous study by our group demonstrated that face-to-face training is the gold standard for teaching these skills. It is unclear if high quality educational videos can augment this teaching.

**Methods:**

Forty-two Medical Students naïve to large joint examination were recruited and block randomised to two groups. The control group had face-to-face teaching alone. The intervention group had their teaching augmented with a custom educational video accessed via a web portal. Participants were assessed on their examination of a large joint using a previously standardised assessment tool at baseline and 7 days post intervention. Assessors were blinded to intervention type.

**Results:**

There was no significant difference in the mean baseline scores. Mean baseline scores were 3.35 (11.2%, SD = 2.2, SE = 0.49) for the face-to-face only group and 2.65 (8.8%, SD = 1.39, SE = 0.31) for the video adjunct group [*p* = 0.137]. There was a significant difference in the improvement in score after intervention between each group [*p* = 0.005]. The mean improvement in score was 15.42 (SD = 5.64, SE = 1.29) for the face-to-face only group and 20.68 (SD = 4.33,SE = 0.99) for the video adjunct group.

**Conclusion:**

When used as an adjunct to more traditional face-to-face teaching methods, a custom-made educational video significantly improves the teaching of clinical examination skills and there is a role for these resources in augmenting traditional teaching methods.

**Supplementary Information:**

The online version contains supplementary material available at 10.1186/s12909-022-03974-8.

## Background

Competent and thorough clinical examination is of vital importance in the care of patients. Good clinical examination skills have been shown to increase the quality of patient care, improve the doctor patient relationship and even reduce the cost of care [1–3].

Therefore, it is disappointing that there is a lack of published evidence looking at the relative effectiveness of physical examination teaching. This situation is compounded by the fact that there are a number of studies have shown that these skills, and in particular skills in musculoskeletal (MSK) examination, are often lacking in undergraduate and postgraduate trainees [1, 4, 5].

MSK teaching is often underrepresented within medical education with studies showing that as little as 2% of time teaching examination skills is focussed on MSK despite MSK problems making up to 20% of presenting complaints within primary care [4]. Poor MSK clinical examination skills teaching is reflected in exam performance with a study by Pietzmann et al. demonstrating that candidates undertaking the USMLE (United States Medical Licensing Examination®) clinical exams performed significantly worse in MSK and neurological examination encounters compared to others [5].

The teaching of MSK examination skills remains widely varied across medical education providers [6]. Given the importance of MSK examination, and the concerns around the teaching of these skills, it is important to establish how best to teach MSK examination. These skills are typically taught through a combination of large group didactic and small group seminar-style sessions but there has been a lack of agreement over the most effective teaching method [1]. In a recent randomised control trial performed by our group (Brewer at al. 2021) it was demonstrated that face-to-face teaching was superior to both text-book learning and video learning in teaching clinical examination of the shoulder joint [7].

An integrative review of papers looking at trends in Medical Education describes how advanced technology is facilitating students’ education by allowing more individualised learning, social interaction between students and their teachers, and access to a wider variety of resources regardless of time or geographical location [8]. There is also emerging evidence for technology-enhanced clinical examination teaching, including online modules and videos [1]. The SARS-Cov-2 pandemic has also led to much more widespread use of electronic educational resources and online learning is now a much bigger part of everyday clinical practice [9–11].

The aim of this study was to establish whether a custom-made educational video augments the face-to-face teaching of clinical examination skills when teaching novice medical students clinical examination of the shoulder joint. We hypothesise that use of a video resource as an adjunct to small group teaching will give superior learning outcomes compared to small group teaching alone.

## Materials and methods

This study was a prospective randomised trial comparing two arms of intervention for teaching clinical examination skills to first and second year medical students with no previous experience of shoulder examination. The two arms were face-to-face teaching alone (F2F) and face-to-face teaching plus access to an online video resource (F2FV).

First and second year medical students at the University of Sheffield with no prior formal clinical examination skills teaching were chosen as the study group. All first and second year students were eligible for inclusion. The only exclusion criteria was previous teaching on MSK examination. Participants from both year groups were grouped together based on having the same limited clinical experience as part of their curriculum and therefore no differences in ability to perform a clinical examination were expected. Recruitment was performed through opportunity sampling with the study proposal being presented to each year group and providing students with an information booklet detailing the aims, objectives and requirements for participation in the study (see Supplementary Materials 1). Participation was voluntary and informed consent was obtained from participants via a signed a consent form (see Supplementary Materials 2). Ethical approval for this study was granted by the University of Sheffield (Reference Number 031696 – see Supplementary Materials 3) and all methods were performed in accordance with the approved study proposal.

We recruited 42 participants to the study. These participants were block randomised to one of the two interventions by a computer random number generator (21 participants per study arm).

**Fig. 1:**
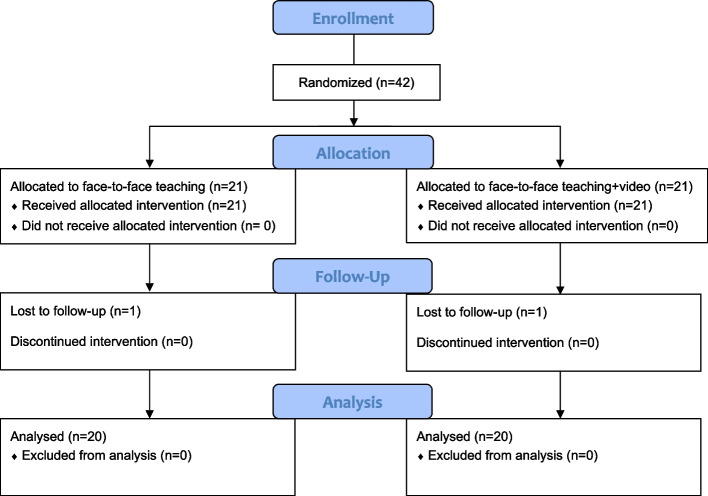
Consort flow diagram showing trial recruitment and retention

54.8% of participants identified as female (*n* = 23) and 45.2% as male (*n* = 19). The overall average age of participants was 20.5 years (19.8 years for face-to-face only group and 20.7 years for the video adjunct group). Figure 1 shows the participant flow through the trial.

On day zero all participants underwent a pre-intervention assessment during which they were asked to examine the shoulder of a volunteer patient with no abnormal pathology. They were assessed against a standardised score sheet which was validated in our previous study [7]. Assessments were performed by senior orthopaedic trainees with relevant expertise in examination of the shoulder. Participants were aware that they would be required to perform a clinical examination but were not informed of the system this would be of.

All participants were then asked to attend a 30 minute face-to-face small group teaching session on clinical examination of the shoulder joint. All sessions were taught by the same senior author to 4 separate groups of 10–12 participants on day zero. Participants were randomly allocated to these groups based on a participant number assigned on recruitment. Participants were taught a standardised method of examining the shoulder based on a previously published technique [12]. The examination was demonstrated on a healthy volunteer.

Those participants randomised to the video adjunct group were emailed access to a custom-made 30-minute online video resource at the same date and time following completion of all face-to-face teaching sessions on day zero. The video resource was produced and edited to a high quality for the purposes of this study with the same senior author, structure and content as the face-to-face teaching sessions. The video included demonstration of the examination technique taught in the face-to-face session with demonstration of these on a model patient from a number of angles. Graphic overlays of anatomical pictures were also included. The process and sequence of shoulder joint examination taught in the video resource mirrored that of the face-to-face seminar. This resource was uploaded to an online platform (SproutVideo LLC) which allowed each candidate to have a personalised and password-protected profile. This platform also allowed advanced analytics including who accessed the video and when, how many times the video was watched, and on what kind of device it was watched.

Those participants in the face-to-face teaching alone group were asked not to access any video material in relation to examination of the shoulder joint during the study period. All participants were advised that they could access and use other study materials as they wished throughout the study period to allow this to be as ‘real-life’ as possible.

At day 7 post-intervention participants were asked to attend for a second assessment. This was performed in the same manner and using the same score sheet as the initial pre-intervention assessment. Assessors were blinded to intervention group and there was a dropout rate of 2 between the pre- and post-intervention assessments.

## Results

The score sheet used had been validated in our initial study with inter-rater reliability of the performance scores indicating strong agreement between assessors when six candidates were independently assessed by two examiners at the same time (Cohen’s kappa, k = .839, *p* < .001) [7]. The score sheet remained unchanged for this study and therefore inter-rater reliability was not re-assessed.

The mean pre-intervention assessment score was 3.00/30 (10%, SD = 1.87, SE = 0.30). For the face-to-face teaching only group the mean pre-intervention assessment score was 3.35/30 (11.2%, SD = 2.2, SE = 0.49) and for the video adjunct group was 2.65/30 (8.8%, SD = 1.39, SE = 0.31).

**Fig. 2:**
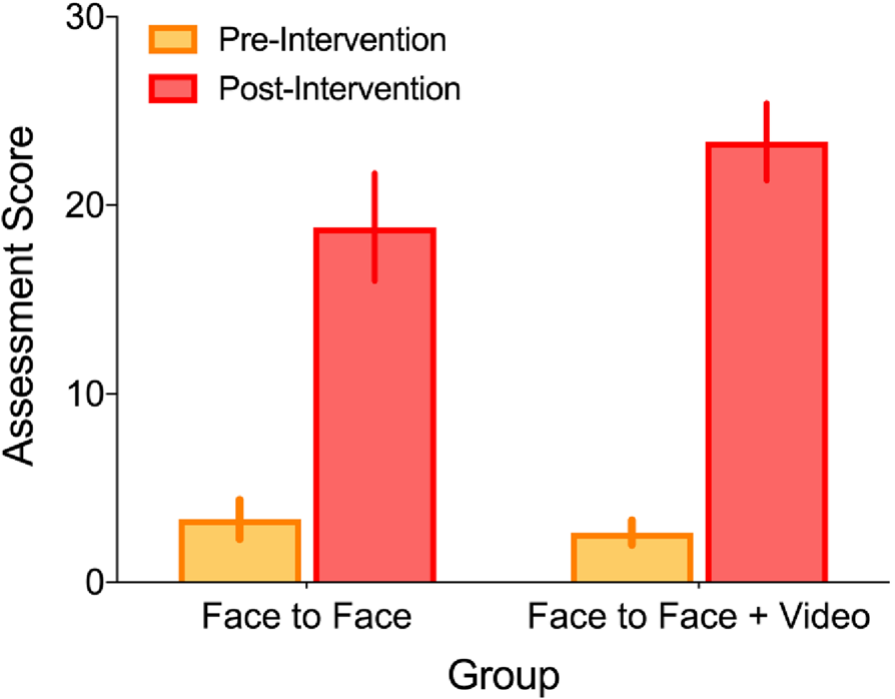
Pre- and post-intervention assessment scores for each of the training groups. Error bars represent 95% confidence intervals

Between the pre- and post-intervention assessments there was a dropout rate of 2 meaning complete data was available for 40 participants (20 participants per study arm). The mean post-intervention assessment score was 21.11/30 (70%, SD = 5.58, SE = 0.88). For the face-to-face teaching only group the mean improvement in score was 15.42 (SD = 5.64, SE = 1.29) and for the video adjunct group this was 20.68 marks (SD = 4.33, SE = 0.99). The pre- and post-intervention assessment scores for each group with 95% confidence intervals are represented in Fig. 2.

To examine whether the training conditions resulted in reliable differences in performance, we computed analysis of covariance (ANCOVA). For the ANCOVA, the fixed factor was the intervention (face-to-face teaching alone vs face-to-face teaching with video adjunct) and the dependent variable was post-intervention assessment score. The pre-intervention assessment score was entered as a covariate to control for individual differences in baseline performance. We considered α < =.05 to be statistically significant and report eta squared (η [2]) to indicate effect sizes. Estimated marginal means adjusted for pre-intervention assessment scores are reported with +/− 1 SEM, where higher scores indicate better performance.

There was no statistically significant influence of the covariate, pre-intervention score [F(1,35) = 2.32, *p* = .137, η^2^ = .05], but we found a statistically significant difference in learning as a function of teaching method [F(1,35) = 9.00, *p* = .005, η^2^ = .194]. The estimated marginal means indicated that participants in the video adjunct group (M = 23.6, SE = 1.17) performed better than those in the face-to-face teaching alone group (M = 18.6, SE = 1.17). When age and gender of participants were added as covariates to this model, we found no effect of either (p’s ≥ .578).

### Video data

**Fig. 3:**
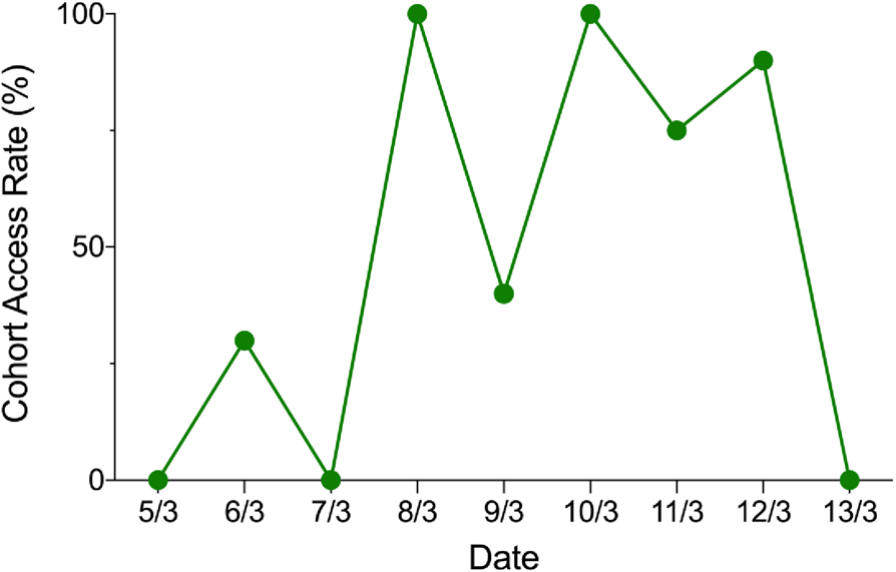
Play rate of the video resource by date for the video adjunct cohort

The web platform we used allowed analytics of how and when the video resource was accessed by participants. 100% of those participants who were given access to the video resource viewed this on at least 1 occasion during the 1-week period between the pre- and post-intervention assessments (Fig. 3).

There was also a mix of devices used to access and play the video resource: 77.8% (42 views) of the time the video resource was accessed on a desktop and 22.2% (12 views) on a mobile device.

## Discussion

Face to face teaching has previously shown to be the gold standard for teaching clinical examination skills [7]. Here, we have shown that it can be enhanced with the use of a purpose made educational video, with statistically significant improvements in assessment scores.

The importance of dialogue and social interaction in teaching and learning has been described throughout historical literature related to education [13]. However, the use of online teaching resources has come to the forefront in recent years and even more so with the increased use of virtual platforms for meetings and education during the covid-19 pandemic [9–11]. 100% of the participants in this study owned a smart phone enabling easy access to wealth of resources relevant to their curriculum, such as the educational video made for this study. With easy access to a huge number of online teaching resources comes the risk of misinformation. The provision of custom-made educational videos by undergraduate and postgraduate educators has the potential to not only enhance teaching but reduce the risk of wasted time and effort for learners in discerning the useful resources from the misleading.

A study looking at the quality of information provided in videos relating to rheumatoid arthritis accessible on YouTube, found that only 50% of videos were ‘useful’, with 30% being classified as ‘misleading’ [14]. Similar work looking at videos on YouTube relating to examination of the cardiovascular and respiratory systems felt that less than 50% of those videos reviewed were educationally useful [15]. Lee et al. (2018) analysed 200 YouTube videos for use as educational tools for teaching special tests in shoulder joint examination. They found that 25% of these videos could be classed as ‘very useful’, 54% as ‘somewhat useful’, but 16% as ‘misleading’ and 5% as ‘not useful’ [16]. Zwerus et al. reviewed 126 widely available videos on elbow examination and found only 23 of these were of potential educational benefit [17].

The cost of poor clinical examination skills should not be underestimated. Increased diagnostic error, false positives and excessive diagnostic tests have both monetary and safety costs to patients and the wider healthcare system [18, 19]. Beyond the clinical expense of poor examination skills teaching are other costs such as that of running an OSCE (Objective Structured Clinical Examination), which can be upwards of £65,000 [20]. Reducing the risk of needing to run repeated OSCE examinations due to candidate failure through improved clinical examination skills teaching has the potential to save money in this area as well.

Cost analysis was not looked at during this study but we estimated that, at the time of creating our video, it would cost approximately £5000 for professional filming and production. This estimate is based on the use of a commercial agency to record and edit a video of this type and quality. This cost is likely to be significantly reduced if institutions have the relevant in-house expertise and technology, something that has become more commonplace since the recent pandemic. Given that examination skills would not be expected to evolve significantly over time, it is unlikely that any initial cost would need to be repeated. There is the potential for a significant return on investment but studies specifically addressing cost effectiveness would be needed.

We made some changes to the design of this study compared to the first in our series. In our original study dominant learning styles were obtained for each participant through use of a ‘VARK’ (Visual, Aural, Read/write, Kinesthetic sensory modalities) questionnaire [21]. On statistical analysis there was no reliable impact on the results based on the participant learning styles [7]. There remains a certain amount of controversy on the usefulness of the VARK questionnaire as, whilst this has been validated [22], there is no evidence to show that changing the method of teaching to align with learning style improves outcomes [23, 24]. Secondly, we chose to assess participants once at day 7 post-intervention compared to day 5 and day 19 as per the initial study. This was done in order to reduce the risk of participant drop out given our study group were full time medical students and because in our previous study there was no difference in performance between the day 5 and day 19 assessments [7].

We recognise that one of the limitations of this study is that longer term retention of information has not been assessed. However, being able to maintain a robust and controlled study design such as this in our study group over a long period of time would not have been realistic. The sample size used here was equivalent to that in our original study but we note that further, larger scale studies are warranted [7]. Other recognised limitations of our study are the bias associated with the motivation of students volunteering to participate in an educational study and the fact that an experienced and enthusiastic teacher can have a significant influence on learning and information retention.

We may consider that the benefit of a video resource for the F2FV intervention group was due to more training thanks to an additional educational resource. However, participants across both intervention groups were permitted to use any other forms of non-video educational material (e.g. textbooks, their own notes from the face-to-face session) during the study period, though the extent to which participants did this was not assessed. Whether repeated face-to-face teaching sessions may offer the same benefit as a video adjunct was also not assessed, however in a real life setting this is likely to be impractical given the wide breadth of curriculum medical schools are required to cover and the increasing number of medical students they are required to teach (the number of spaces available in UK medical schools in 2021 was 9500 but this is predicted to increase further as the demand for more doctors within the NHS also increases) [25]. A video resource mirroring a face-to-face teaching session is a useful adjunct for allowing repeated exposure to the same learning material without the need for repeated effort, time & resources for educators. One future avenue to explore is the relationship between the frequency and nature of participant engagement with video resources with learning rates, which could help support the development and refinement of future content.

This is now the second study in our series looking at the relative effectiveness of different teaching modalities in isolation and combination when teaching physical examination skills. To our knowledge this is the only randomised control trial assessing the use of a custom-made educational video as an adjunct to more traditional methods for clinical examination teaching. Major strengths of this study include the robust methodology, with adherence to study protocols for each participant, and blind assessment at all stages.

Virtual learning in its many forms (ranging from video material to virtual and augmented reality [26, 27]) has several potential benefits for learners, including the potential for increased recall and retention with combined experiences, the reduced risk of burnout due to travelling for teaching sessions, 24/7 access to relevant and accurate information, and the improvement in skills in managing technology [9]; the latter being particularly relevant in our developing healthcare system. It is clear that virtual teaching modalities can enrich a learner’s experience and the pandemic has given educators the opportunity to develop a more blended learning environment.

## Conclusion

A high quality custom-made educational video can augment the teaching of shoulder examination in face-to-face teaching sessions. It is important to create electronic resources to complement normal teaching. We do not support the use of electronic resources as a replacement for face-to-face teaching.

## Supplementary Information

**Additional file 1.**

**Additional file 2.**

**Additional file 3.**

## Data Availability

The datasets used and analysed during the current study are available from the corresponding author on reasonable request.

## Abbreviations

MSK: Musculoskeletal
ANCOVA: Analysis of covariance
USMLE: United States Medical Licensing Examination®
OSCE: Objective Structured Clinical Examination
VARK: Visual, Aural, Read/write, Kinesthetic sensory modalities.
