# Supplementary material for: Can educational video resources improve learning when used to augment traditional teaching of clinical examination? A randomized control trial of novice medical students
Source: BMC Med Educ. 2023 Jan 12;23:21. doi: 10.1186/s12909-022-03974-8 (PMC9834676; doi:10.1186/s12909-022-03974-8)
Supplement: Supplementary file 1 — Additional file 1. [file 12909_2022_3974_MOESM1_ESM.docx]

Clinical Examination Study – participant information sheet

Introduction:

You are being invited to take part in a research project. Before you decide it is important for you to understand why the research is being done and what it will involve. Please take time to read the following information carefully and discuss it with others if you wish. Ask us if there is anything that is not clear or if you would like more information. Take time to decide whether or not you wish to take part. Thank you for reading this.

Further Information

This study has been designed to assess whether a custom-made educational video can improve learning when used to augment traditional teaching of clinical examination skills. The study will involve two standardised assessments that will take 10 minutes each, and one face-to-face teaching session of 30 minutes. The assessments and teaching session will all happen within a 7 to 14 day period.

Time commitment

The total time needed for the study will be about 90 minutes plus travel time to the medical school over a 7-14 day period.

Why have I been chosen?

All first year students at Sheffield University medical school have been offered the chance to participate. We are hoping to recruit 70 or more participants in total.

Do I have to take part?

Participation in this study is entirely voluntary and there will be no negative consequences of not participating in this study. You can withdraw from the study at any time without adverse effect by contacting one of the study team.

What will happen if I take part?

If you decide to take part in this study you will be asked to attend the medical school for a baseline assessment. This will take a maximum of 15 minutes (excluding travel time). You will be randomised to one of the two arms of the study – either a face-to-face teaching session on major joint examination or the face-to-face teaching session and access to a custom made online video. You will be re-assessed at 1 and 7 days after the teaching session.

Will I be recorded and how will the recorded media be used?

No you will not be recorded.

Disadvantages and risks:

We do not anticipate any risk to participants but you will be assessed which some people find a stressful experience. The assessment is to assess the teaching methods, and is not an assessment of your ability. The study will not impact on your academic progression and the results will not form part of your degree

Advantages and benefits:

By participating in this study you will receive a tailored teaching session on examination of a major joint. You will also help us assess whether instructional videos are a useful teaching method, and assess whether they are acceptable to students, which will potentially benefit both undergraduate and postgraduate teaching. There will be a potential benefit to participants of learning major joint examination to a standard over and above the regular curriculum.

Legal basis of data processing

*According to data protection legislation, we are required to inform you that the legal basis we are applying in order to process your personal data is that ‘processing is necessary for the performance of a task carried out in the public interest’ (Article 6(1)(e)). Further information can be found in the University’s Privacy Notice* [*https://www.sheffield.ac.uk/govern/data-protection/privacy/general*](https://www.sheffield.ac.uk/govern/data-protection/privacy/general)*.*

What will happen to the data collected, and the results of the research project?

The data collected will be anonymised, stored electronically and may be submitted for publication in a peer reviewed journal and presented at national and international meetings. It will not be possible to identify the participants in any of these formats. The work will not be presented locally without the agreement of all study participants. No data will be transferred outside the EEA.

Due to the nature of this research it is possible that other researchers may find the data collected to be useful in answering future research questions. We will ask for your explicit consent for your data to be shared in this way.

All captured data will be destroyed once this study has been completed, and the data is not needed for further evaluation of this work.

Who is organising this research?

James Tomlinson (Orthopaedic Consultant) and Ellie Flatt (Orthopaedic Registrar) conducting this research.

Who is the Data Controller?

The University of Sheffield will act as the Data Controller for this study. This means that the University is responsible for looking after your information and using it properly.

Who has ethically reviewed the project?

This project has been ethically approved via the University of Sheffield’s Ethics Review Procedure, as administered by the Department of Medical Education.

What if something goes wrong and I want to make a complaint?

If you wish to make a complaint you can contact the project lead via e-mail [jamestomlinson1@nhs.net](mailto:jamestomlinson1@nhs.net) If this does not resolve the issue you can contact the Academic Lead for clinical skills, Mr Amir Burney via e-mail [a.burney@sheffield.ac.uk](mailto:e.a.wood@sheffield.ac.uk) If the complaint relates to how your personal data has been handled, information about how to raise a complaint can be found here in the University’s privacy notice: <https://www.sheffield.ac.uk/govern/data-protection/privacy/general>

Contact for further information:

For further information about the study please contact

Principal Investigator James Tomlinson [jamestomlinson1@nhs.net](mailto:jamestomlinson1@nhs.net)

Study co-ordinator Ellie Flatt eflatt@doctors.org.uk
