# Supplementary material for: Can educational video resources improve learning when used to augment traditional teaching of clinical examination? A randomized control trial of novice medical students
Source: BMC Med Educ. 2023 Jan 12;23:21. doi: 10.1186/s12909-022-03974-8 (PMC9834676; doi:10.1186/s12909-022-03974-8)
Supplement: Supplementary file 2 — Additional file 2. [file 12909_2022_3974_MOESM2_ESM.docx]

**Clinical Examination Study Consent Form**

| ***Please tick the appropriate boxes*** | **Yes** | **No** |
| --- | --- | --- |
| **Taking Part in the Project** |  |  |
| I have read and understood the project information sheet dated 08/11/2019 or the project has been fully explained to me. (If you will answer No to this question please do not proceed with this consent form until you are fully aware of what your participation in the project will mean.) |  |  |
| I have been given the opportunity to ask questions about the project. |  |  |
| I agree to take part in the project. I understand that taking part in the project will include two assessments and a teaching session. The assessments will not affect my medical school marks and will not be shared with the medical school. |  |  |
| I understand that my taking part is voluntary and that I can withdraw from the study at any time; I do not have to give any reasons for why I no longer want to take part and there will be no adverse consequences if I choose to withdraw. |  |  |
| **How my information will be used during and after the project** |  |  |
| I understand my personal details such as name, phone number, address and email address etc. will not be revealed to people outside the project. |  |  |
| I understand and agree that my marks may be quoted in publications, reports, web pages, and other research outputs. I understand that I will not be named in these outputs. |  |  |
| I understand and agree that other authorised researchers will have access to this data only if they agree to preserve the confidentiality of the information as requested in this form. |  |  |
| I understand and agree that other authorised researchers may use my data in publications, reports, web pages, and other research outputs, only if they agree to preserve the confidentiality of the information as requested in this form. |  |  |
| I give permission for the examination scores, and candidate number data that I provide to be deposited in Sheffield Teaching Hospitals so it can be used for future research and learning |  |  |
| **So that the information you provide can be used legally by the researchers** |  |  |
| I agree to assign the copyright I hold in any materials generated as part of this project to The University of Sheffield. |  |  |

|  |  |  |
| --- | --- | --- |
| Name of participant [printed]  Email address of participant | Signature | Date |
|  |  |  |
| Name of Researcher [printed] | Signature | Date |
|  |  |  |

**Project contact details for further information:**

James Tomlinson (Principle Investigator) [jamestomlinson1@nhs.net](mailto:jamestomlinson1@nhs.net)

Ellie Flatt (Study Co-ordinator) [eflatt@doctors.org.uk](mailto:eflatt@doctors.org.uk)
