# Supplementary material for: Can educational video resources improve learning when used to augment traditional teaching of clinical examination? A randomized control trial of novice medical students
Source: BMC Med Educ. 2023 Jan 12;23:21. doi: 10.1186/s12909-022-03974-8 (PMC9834676; doi:10.1186/s12909-022-03974-8)
Supplement: Supplementary file 3 — Additional file 3. [file 12909_2022_3974_MOESM3_ESM.pdf]

Downloaded: 30/01/2020
Approved: 27/01/2020

James Tomlinson
Medical School

Dear James

PROJECT TITLE: Randomised trial of face to face teaching vs. face to face plus video teaching to teach shoulder examination
APPLICATION: Reference Number 031696

On behalf of the University ethics reviewers who reviewed your project, I am pleased to inform you that on 27/01/2020 the above-named project was **approved** on ethics grounds, on the basis that you will adhere to the following documentation that you submitted for ethics review:

- University research ethics application form 031696 (form submission date: 21/12/2019); (expected project end date: 31/03/2020).
- Participant information sheet 1072354 version 2 (21/12/2019).
- Participant consent form 1072355 version 2 (21/12/2019).

If during the course of the project you need to deviate significantly from the above-approved documentation please inform me since written approval will be required.

Your responsibilities in delivering this research project are set out at the end of this letter.

Yours sincerely

Melanie Viphakone
Ethics Administrator
Medical School

Please note the following responsibilities of the researcher in delivering the research project:

- The project must abide by the University's Research Ethics Policy: https://www.sheffield.ac.uk/rs/ethicsandintegrity/ethicspolicy/approval-procedure
- The project must abide by the University's Good Research & Innovation Practices Policy: https://www.sheffield.ac.uk/polopoly_fs/1.671066!/file/GRIPPolicy.pdf
- The researcher must inform their supervisor (in the case of a student) or Ethics Administrator (in the case of a member of staff) of any significant changes to the project or the approved documentation.
- The researcher must comply with the requirements of the law and relevant guidelines relating to security and confidentiality of personal data.
- The researcher is responsible for effectively managing the data collected both during and after the end of the project in line with best practice, and any relevant legislative, regulatory or contractual requirements.
